# Vinyl ethers and epoxides photoinduced copolymerization with perfluoropolyalkylether monomers

**DOI:** 10.1007/s00396-020-04723-3

**Published:** 2020-09-09

**Authors:** Giuseppe Trusiano, Alessandra Vitale, Céline Bonneaud, Diego Pugliese, Sara Dalle Vacche, Christine Joly-Duhamel, Chadron M. Friesen, Roberta Bongiovanni

**Affiliations:** 1 Department of Applied Science and Technology, Politecnico di Torino, Corso Duca degli Abruzzi 24, 10129 Torino, Italy; 2 Institut Charles Gerhardt Montpellier, University of Montpellier, CNRS, ENSCM, Cedex 5, 34296 Montpellier, France; 3 Department of Chemistry, Trinity Western University, 7600 Glover Road, V2Y 1Y1 Langley, BC Canada

**Keywords:** Perfluoropolyalkylethers, Cationic polymerization, UV curing, Sliding angle, Refractive index

## Abstract

New perfluoropolyalkylether (PFPAE) monomers, chain extended with different alkyl groups and functionalized with vinyl ether or epoxide end-groups, were employed, together with trimethylolpropane trivinyl ether or trimethylolpropane triglycidyl ether, to produce fluorinated copolymers. The photoinduced cationic polymerization was investigated, and the PFPAE-based copolymer properties were thoroughly characterized. Interesting surface properties and two different values of refractive index were observed: thus, these fluorinated copolymers can be suitable materials for the manufacture of self-cleaning coatings and optical waveguides.

**Electronic supplementary material:**

The online version of this article (10.1007/s00396-020-04723-3) contains supplementary material, which is available to authorized users.

## Introduction

The photoinduced cationic polymerization of vinyl ether or epoxy-based formulations is nowadays a well-established process, which owes its development and implementation in several industrial fields, such as inks, adhesives, coatings, and electronics, to its significant advantages: the photoinduced process is fast, does not require either solvent or heating, there is no inhibition by oxygen, products show low shrinkage, good mechanical and adhesion properties [1–4].

The design of new monomers suitable for cationic photopolymerization processes is therefore interesting. In this context, perfluoropolyalkylethers (PFPAEs) are attractive building blocks. They are non-toxic [5–8] and show exceptional properties: high thermal, chemical, corrosion, and weather resistances, very low refractive index, surface tension, glass transition temperature, adhesion, and friction [9–12]. Therefore, they have found applications as lubricants, surfactants, and finishing agents. Several PFPAE structures exist: they are made up of one or two of the following repetitive units: –(CF_2_O)–, −(CF_2_CF_2_O)–, −(CF_2_CF_2_CF_2_O)–, and –(CF(CF_3_)CF_2_O)–. CF_3_O–, C_2_F_5_O–, C_3_F_7_O–, and COFC_2_F_4_– are the end-groups, depending on the synthesis route [9, 11]. These end-groups can be functionalized in a large variety of ways, as described in several reviews [9]. In the past years, an increasingly growing number of attempts to functionalize PFPAEs with photoreactive terminal groups, exploitable as monomers/oligomers for preparing polymers via photopolymerization reactions have also been observed [9, 13–15]. The UV-cured fluorinated copolymers obtained, mostly acrylic, have been proposed as fouling-release coatings, microfluidic devices, non-flammable polymeric electrolytes for lithium ion batteries, microlithography resins, etc. [13, 14, 16, 17]. A few examples of PFPAE monomers bearing allylic and epoxy groups are also reported [18].

In our recent works, we showed that a very low amount (< 5 wt%) of monofunctional PFPAE epoxides photocopolymerized with common epoxides guaranteed a significant modification of the surface properties [13, 14, 19–22], even more effective than fluorinated vinyl ethers and epoxides used previously, which pose safety issues due to the long fluoroalkylic chain [20, 23].

In this work, we describe the cationic photocopolymerization of new difunctional PFPAE vinyl ethers and epoxides with traditional non-fluorinated monomers, using a relatively high PFPAE comonomer content (25 wt%). The photopolymerization reaction of the fluorinated copolymers was studied, and their surface and bulk properties have been characterized as well.

Biphasic morphologies have been obtained resulting into a surface segregation of the PFPAE comonomers and into peculiar optical properties so that these materials can be suitable for applications as high-performance coatings and polymer optical waveguides.

## Materials and methods

### Materials

**Fig. 1:**
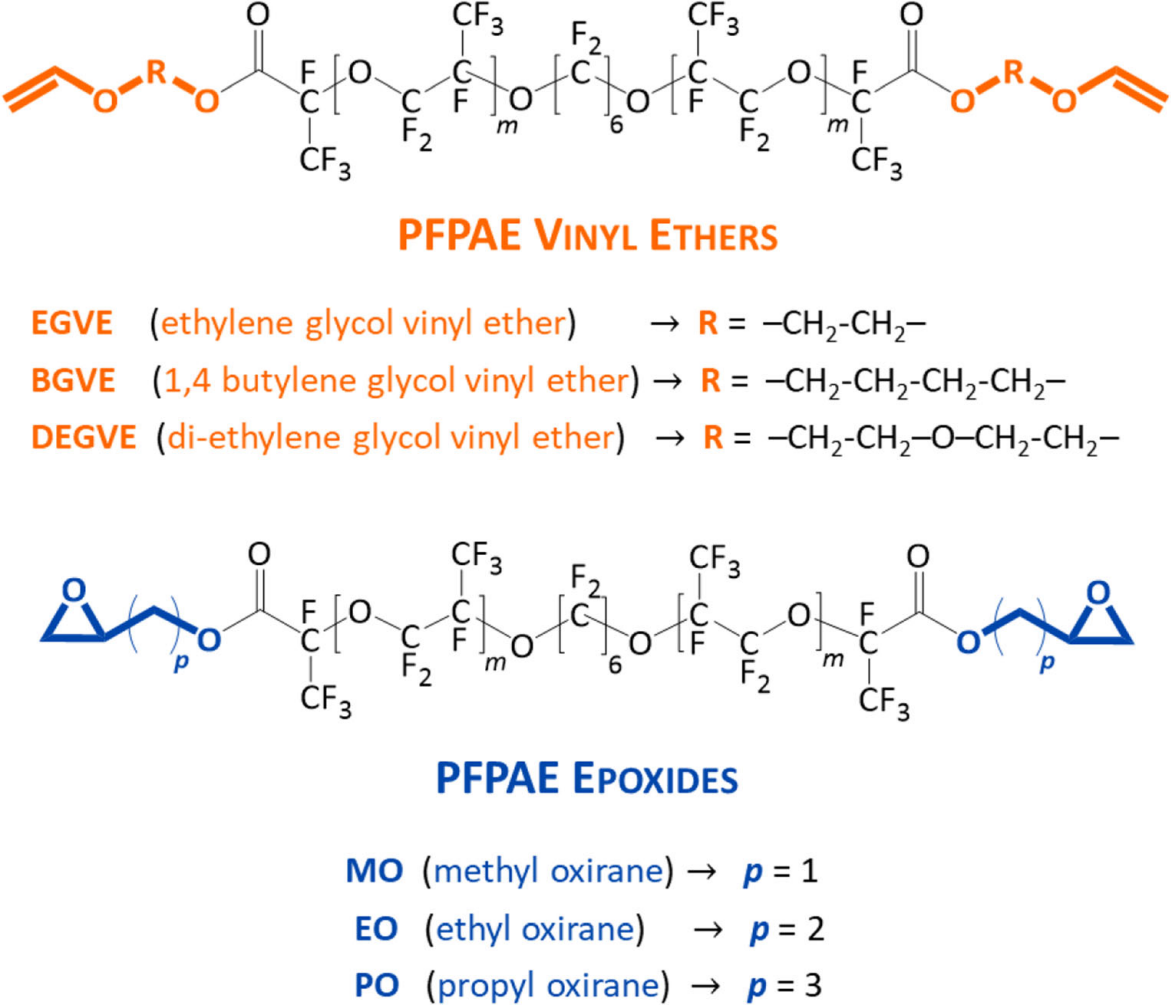
PFPAE comonomers used in the UV-induced copolymerization with non-fluorinated monomers. The values of the repeat units (*m*) can be found in Table 1

The difunctional fluoromonomers employed in this work are reported in Fig. 1.

They were synthesized starting from PFPAE diacyl fluoride or dicarboxylic acid. These products were chain extended with alkyl groups and functionalized with vinyl ether and epoxide end-groups: monofunctional products were also present. The average functionality, estimated by ^19^F-NMR analyses, is reported in Table 1. Details about the synthesis, functionalization, and characterization of the functional PFPAE derivatives used in this work can be found in a forthcoming paper [24].

**Table 1:** Number of repeat units, average molecular weight (M_*n*_), difunctional content, and average functionality (from ^19^F-NMR spectra) of the functionalized PFPAE monomers

System	*m*	PFPAE average M_*n*_ (g/mol)	PFPAE difunctional content (mol%)	PFPAE average functionality
PFPAE-EGVE	6	1740	56	1.56
PFPAE-BGVE	8	2130	88	1.88
PFPAE-DEGVE	7	2000	43	1.53
PFPAE-MO	12	2720	63	1.63
PFPAE-EO	8	2130	41	1.41
PFPAE-PO	10	2530	58	1.58

The hydrogenated (i.e., non-fluorinated) comonomers used are trimethylolpropane trivinyl ether (TVE, kindly provided by BASF Germany) and trimethylolpropane triglycidyl ether (TGE, purchased by Sigma-Aldrich). Triphenylsulfonium hexafluorophosphate salts, 50 wt% in propylene carbonate, purchased from Sigma-Aldrich (Italy), was used as the cationic photoinitiator.

### Photoinduced polymerization

The UV-reactive formulations were prepared by mixing 75 wt% of the hydrogenated resin and 25 wt% of the PFPAE monomer. In each formulation, 2 wt% of the photoinitiator was added. The details of the formulations used in this work are collected in Table 2.

**Table 2:** Composition details and functionality of the different investigated UV-reactive systems

Sample	PFPAE monomer (wt%)	Resin (wt%)	PFPAE/resin molar ratio	Average functionality
TVE	–	100	0.00	3.00
PFPAE-EGVE + TVE	25	75	0.04	2.94
PFPAE-BGVE + TVE	25	75	0.03	2.96
PFPAE-DEGVE + TVE	25	75	0.03	2.95
TGE	–	100	0.00	3.00
PFPAE-MO + TGE	25	75	0.04	2.95
PFPAE-EO + TGE	25	75	0.05	2.93
PFPAE-PO + TGE	25	75	0.04	2.94

The UV-sensitive mixtures were coated onto a glass substrate, using a wire-wound applicator. The glass substrate used was a microscope slide (pre-cleaned/ready-to-use) purchased from Thermo-Scientific, used as received without any surface treatment.

The samples were irradiated by means of a high-pressure mercury arc lamp Dymax ECE (predominately producing UV-A light (400–320 nm) and some amount of UV-B light (320–280 nm)) [25], using a total light intensity of 150 mW/cm^2^ for 5 min. Samples with different thickness, ranging from 100 to 300 μm, were prepared. Light intensity was measured with a UV Power Puck II digital radiometer.

After irradiation, the samples had been stored for at least 24 h at room temperature before properties were evaluated. This wait time allowed for complete dark post-curing reactions, which are typical of a cationic process.

### Polymer characterization

The conversion of the photopolymerization reaction was monitored by Fourier transform-infrared (FT-IR) spectroscopic analysis and by photodifferential scanning calorimetry (photo-DSC).

Real-time FT-IR spectroscopic analyses were performed using a Nicolet iS50 FT-IR spectrometer (Thermo Fisher Scientific). Simultaneously with the FT-IR scan acquisition in transmission mode, resin thin films (i.e., about 10 μm on a Si wafer as substrate) of the reactive monomeric mixtures were irradiated with an UV Hamamatsu LC8 lamp, provided of an optical fiber, having an intensity equal to 100 mW/cm^2^. Light intensity was measured with an UV Power Puck II digital radiometer.

Polymerization conversion was followed by monitoring the decrease of the absorbance of the vinyl ether groups at 1620 cm^−1^ or of the epoxy groups in the region 900–920 cm^−1^ as a function of the irradiation time.

FT-IR spectra were also recorded in attenuated total reflectance (ATR) mode, employing a Nicolet Smart iTX accessory equipped with an ATR diamond crystal, to check the final monomer-to-polymer conversion at the polymer surface after dark-curing (i.e., 24 h at room temperature).

Polymerization kinetics was also studied by photo-DSC analysis, estimating the heat of the photopolymerization reactions by means of a Mettler-Toledo DSC1 STARe System, appropriately equipped with a Hamamatsu LC8 lamp with two beams, one for the sample and the other for the reference, as reported in previous works [26, 27]. To ensure equal illumination conditions throughout the sample volume, about 5 mg of each formulation were cured in open aluminum pans. The sample was extensively flushed with nitrogen. Two scans were performed on each sample to subtract the thermal effect of UV irradiation from the photocuring experiment, each one consisting of 4 min of conditioning at *T* = 25 °C, 20 min of irradiation, and 4 min without UV light. The measurements were carried out with a light intensity of 25 mW/cm^2^ and at room temperature, the increase of temperature due to the irradiation of the lamp being less than 1 °C at the end of the reaction. The reaction was considered completed when it was no longer possible to detect a change in heat flux. The total heat of reaction was then calculated by integrating the area under the exothermic peak [28]. The conversion (*χ*) over time was calculated by Eq. (1):

1 $$ \chi =\frac{\underset{0}{\overset{t}{\int }}\frac{\mathrm{d}H}{\mathrm{d}t}\ \mathrm{d}t}{\Delta {H}_{\mathrm{theor}}} $$

where d*H*/d*t* is the heat flow released by the sample during the photocuring and Δ*H*_theor_ is estimated considering the heat of reaction of 60 and 84 kJ/mol for the polymerization of the epoxy [29] and vinyl-ether monomers [30], respectively.

The insoluble fraction of the crosslinked samples (gel content) was evaluated using the weight loss of the network after a 24 h extraction by 1:1 dichloromethane:pentafluorobutane (DCM:PFB) solution (expressed in weight fraction) at room temperature (ASTM D2765-16) [31]. The crosslinked fraction was then calculated by dividing the mass of the dry sample after extraction by the mass of the original sample.

The thermal stability was determined by thermogravimetric analysis (TGA) using a Mettler Toledo TGA/SDTA-851 instrument. Approximately 10 mg of the sample were placed in an alumina crucible and heated from room temperature to 800 °C under inert atmosphere (60 mL/min), with a heating rate of 10 °C/min.

Differential scanning calorimetry (DSC) thermograms were recorded using a Mettler-Toledo DSC1 STARe System from − 100 to 50 °C using a heat/cool/heat method at a heating and cooling scanning rates of 10 °C/min, under nitrogen flux. The glass transition temperature (*T*_g_) was determined using the midpoint of the heat capacity jump on the second heating cycle thermogram.

To evaluate the copolymers wettability, static contact angle measurements were performed with a FTA 1000C instrument, equipped with a video camera and image analyzer, at room temperature with the sessile drop technique. At least three measurements were performed on each sample and the values averaged. The probe liquids were water and hexadecane, whose surface tensions are 72.1 and 28.1 mN/m, respectively. The surface energy was calculated starting from the contact angle values by the Owens-Wendt’s geometric mean method [32].

Contact angle was also estimated by dynamic measurements: they were performed by increasing the drop volume in the wetting process (advancing contact angle) and then decreasing it in the de-wetting phase (receding contact angle). The syringe needle remained in the drop during the whole process. In the first wetting phase, a 5 μL drop was formed on the solid surface and then slowly increased in volume, reaching a maximum volume of 15 μL. In the second phase, the surface was de-wetted and the drop size reduced. The whole cycle was repeated three times at 1 μL/s, with a delay time of 1 s between each cycle. The contact angle hysteresis (CAH) was calculated as the difference between the measured advancing and receding contact angles.

Sliding angle measurements [33] were performed using the same FTA 1000C apparatus, equipped with a tilting stage. After placing a 20 μL liquid drop on the test surface, the film was tilted at 0.5°/s.

The refractive index (*n*) of the polymeric films was measured at two different wavelengths (633 and 825 nm) by prism-coupling technique (Metricon, model 2010) [34–36]. Ten scans were performed for each wavelength. Estimated error of the measurement was ± 0.001.

## Results and discussion

The difunctional PFPAE monomers with vinyl ether and epoxide end-groups were copolymerized with trifunctional hydrogenated resins exploiting the UV-induced cationic polymerization.

The UV-reactive formulations were prepared by mixing 75 wt% of the hydrogenated resin and 25 wt% of the PFPAE monomer: this concentration was chosen evaluating the compatibility between the hydrogenated and the fluorinated comonomers with the varying of the PFPAE content in the photocurable mixtures. Among the many compositions investigated, amounts of fluorinated comonomers higher than the 25 wt% gave a macroscopic phase-separation at the liquid state as they were highly immiscible with the hydrogenated resins. Whereas, formulations containing 25 wt% of PFPAEs ensure the formation of an UV-cured network whose properties are the combination of those exhibited by the PFPAE-based comonomers with the ones displayed by the hydrogenated comonomers.

**Fig. 2:**
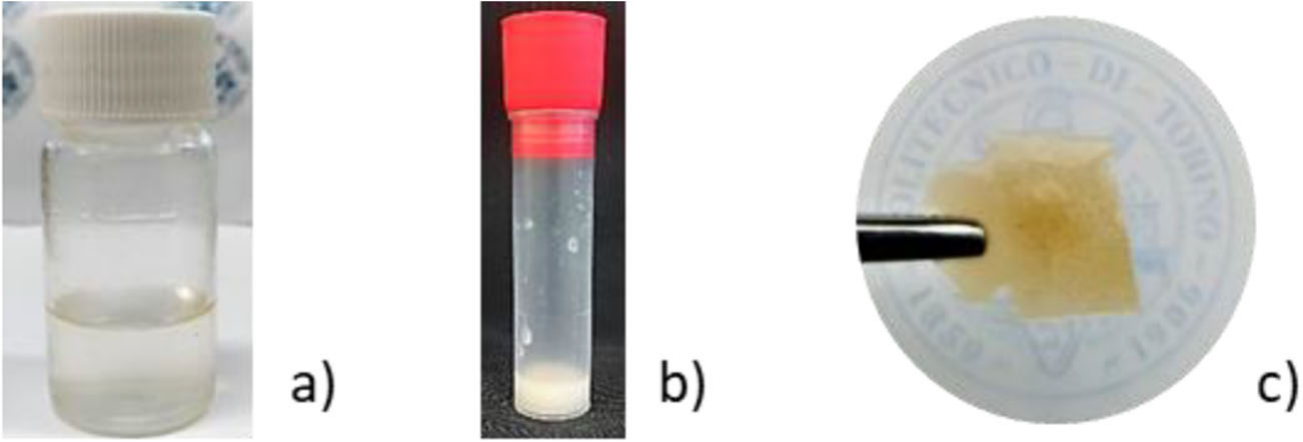
Pictures of **a** PFPAE monomer, **b** photocurable mixture before irradiation, and **c** photocrosslinked copolymer film

The PFPAE comonomers were transparent (Fig. 2a) although biphasic at molecular level since their structure containing a PFPAE chain is linked to alkyl-spacers bearing hydrogenated reactive end-groups [37]. The formulations containing 25% of PFPAEs appeared whitish (Fig. 2b): this heterogeneity is also reflected in the photocured copolymers. Indeed, a microscale phase separation occurs between the fluorinated and the hydrogenated phases, the latter formed by the non-fluorinated resins and the hydrogenated segments of the PFPAE products. Therefore, the photoinduced cationic copolymerization is expected to take place in the hydrogenated domains: it ensured the formation of a solid UV-cured network, colored at a visual inspection (Fig. 2c), as what often happens due to the high exothermicity of the cationic polymerization reaction. Morphological features of the systems will be discussed later in the paper.

It is important to notice that the use of low molecular weight trifunctional hydrogenated comonomers (i.e., TVE and TGE) allowed to significantly improve the mechanical properties showed by the fluorinated homopolymers [24]. In fact, after photocuring, the PFPAE homopolymers are highly viscous semi-solid materials and cannot be used as engineering materials (e.g., coatings). Whereas, the investigated copolymers appear as tight-crosslinked solid networks once UV cured (Fig. 2c).

The photopolymerization kinetics was studied through real-time FT-IR, monitoring the decrease of the intensity of the band of the double bond (1620 cm^−1^) for the vinyl ether copolymers, and of the band of the oxirane ring (900–920 cm^−1^) for the epoxide copolymers, during the UV light exposure of the sample.

**Fig. 3:**
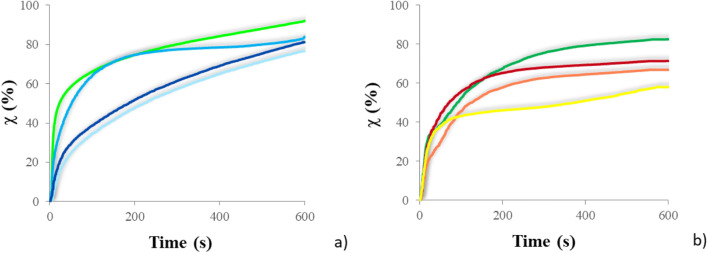
Real-time FT-IR conversion curves of the different systems. **a**  TVE,  PFPAE-EGVE + TVE,  PFPAE-BGVE + TVE, and  PFPAE-DEGVE + TVE; **b**  TGE,  PFPAE-MO + TGE,  PFPAE-EO + TGE, and  PFPAE-PO + TGE

**Fig. 4:**
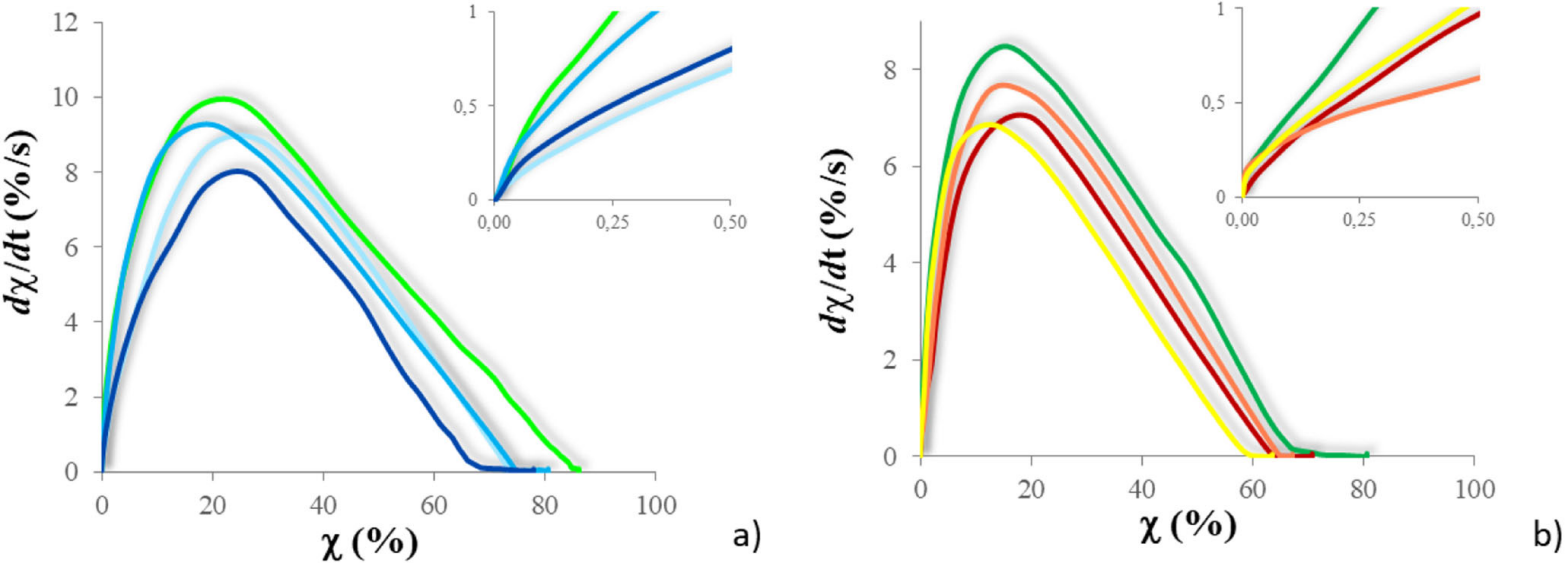
First derivative of the photopolymerization conversion (d*χ*/d*t*) versus conversion (*χ*) for the different systems, by photo-DSC. **a**  TVE,  PFPAE-EGVE + TVE,  PFPAE-BGVE + TVE, and  PFPAE-DEGVE + TVE; **b**  TGE,  PFPAE-MO + TGE,  PFPAE-EO + TGE, and  PFPAE-PO + TGE

The conversion curves of the PFPAE copolymers are reported in Fig. 3 and compared with the homologous homopolymers obtained from TVE and TGE. The kinetic behavior of the PFPAE-based copolymers was also studied using the photo-DSC technique [16, 28]. The curves representing the first derivative of the conversion as a function of time (that is the polymerization rate) are plotted in Fig. 4 as a function of conversion for the vinyl ether (a) and the epoxy systems (b). Table 3 reports the final conversion values measured by photo-DSC analysis and estimated by real-time FT-IR spectroscopy.

As suggested by both FT-IR (Fig. 3) and photo-DSC (Fig. 4) data, the curves feature the kinetic regimes and the complex mechanisms characterizing the photopolymerization reactions of multifunctional systems, as discussed in the literature [2, 38–42]. Although the average functionality is not the same for all reactive systems, ranging from 2.93 to 3.00 (see Table 2), one can suggest that the presence of the PFPAE monomers reduces the reactivity; however, all systems reach more than 40% conversion in less than 3 min (Fig. 3).

Observing Fig. 4 in particular, it is clear that vinyl ethers are more reactive than epoxides; the rate until the maximum of the kinetic curves is higher for the vinyl ether systems than the epoxides. The major reactivity of the vinyl ether copolymers compared with the epoxy copolymers is also confirmed by the FT-IR data about the degree of conversion reached after 10 min of irradiation: the copolymers with vinyl ether functionalities reach conversion values of around 80% (Fig. 3a; Table 3), while those functionalized with epoxy rings reach conversion degrees of around 70% (Fig. 3b; Table 3). The higher reactivity of vinyl ether monomers can be first attributed to the presence of electron rich carbon–carbon double bonds and to the stabilization by resonance of carbocations formed during the propagation. Moreover, the lower *T*_g_ of the polymerized vinyl ether networks (data shown below) helps to reduce the retarding effects caused by the matrix vitrification that occurs when the *T*_g_ of the polymerizing network reaches the curing temperature. As the glass transition of the epoxides is about 10 °C above the one of the vinyl ether monomers, the vitrification causes an anticipated stop of the polymerization reaction in the former case: the diffusion of the unreacted epoxy chains and monomers is inhibited and they remain trapped within the glassy network [2, 43–47].

**Table 3:** Photopolymerization study of the UV-cured copolymers: conversion (*χ*) values obtained by three different techniques (i.e., photo-DSC, real-time FT-IR, FT-IR ATR) and insoluble fraction values

Sample	*χ* (%)			Insoluble fraction (%)
	Photo-DSC^a^	FT-IR^b^	FT-IR ATR^c^	Gel content^d^
TVE	86	92	97	92
PFPAE-EGVE + TVE	74	77	94	82
PFPAE-BGVE + TVE	82	84	98	93
PFPAE-DEGVE + TVE	78	81	96	90
TGE	81	83	98	94
PFPAE-MO + TGE	63	58	82	88
PFPAE-EO + TGE	67	67	85	86
PFPAE-PO + TGE	71	73	93	93

^a^Value of the plateau in the conversion curves with an UV-light intensity of 25 mW/cm^2^ and an irradiation time of 20 min

^b^Value of the plateau in the conversion curves with an UV-light intensity of 100 mW/cm^2^ and an irradiation time of 10 min

^c^Value determined by single spectra taken before and after 24 h from the irradiation (5 min at 150 mW/cm^2^)

^d^Value determined on samples after 24 h from the irradiation (5 min at 150 mW/cm^2^), after 24 h of extraction by 50/50 DCM/PFB at room temperature

As mentioned above, the conversion is lower for the copolymers than for the hydrogenated homopolymers, also because there is a slight reduction of functionality. However, comparing the different copolymerization curves of Figs. 3 and 4, it seems that the length of the hydrogenated spacer between the PFPAE chains and the reactive end-groups of the fluorinated comonomers has an influence on reactivity. Similar behaviors were observed and studied for both living cationic [23, 40, 48] and radical [49, 50] photopolymerization. This effect was observed in the photoinduced polymerization of the macromonomers studied in [24]: the long-fluorinated chain decreased the monomer reactivity by an electron withdrawing effect, lowering the nucleophilicity of the functional group. Therefore, the rate constant of the reaction, the gel point, and the final conversion were found dependent on the distance between the fluorinated segment and the reactive vinyl ether/epoxide groups. In our case, a clear correlation between reactivity and structural parameters is not present: probably, phase separation phenomena have an influence on the photopolymerization behavior of the copolymers [49, 51]. As the length of the alkyl spacing segments decreases, a greater segregation between the hydrogenated and the fluorinated monomers can occur.

The degrees of conversion of the cured products in the form of films were measured by ATR FT-IR analyses, 24 h after the end of the irradiation (Table 3; Figs. S1–S6 of the Supporting Information). The conversion values are quite higher than the final values estimated at the end of the irradiation both by real time FT-IR and photo-DSC: the ATR FT-IR results confirm the presence of the spontaneous “dark curing” reaction exhibited by the cationic photopolymerization [1, 44, 52]. Interestingly, the results obtained by the ATR FT-IR measurements (Table 3) suggest that the longer the hydrogenated spacer between the PFPAE chain and the reactive end-groups of the fluorinated comonomers, the higher the degree of conversion.

The insoluble fraction of the photocured networks was generally good (gel content > 80%, Table 3): these results prove that the photocured networks own good chemical stability towards halogenated solvent. However, the gel content of the copolymers was slightly lower than the one exhibited by the hydrogenated systems due to the lower conversion. Interestingly, the photocured copolymers showed a good dimensional stability: in fact, after 24 h of solvent extraction, a negligible swelling of the copolymers was detected (generally < 5%).

Static contact angle measurements (Table 4) were performed on the UV-cured copolymers to evaluate their wettability with water (polar solvent) and hexadecane (non-polar solvent).

**Table 4:** Static contact angle (*θ*), surface energy (*γ*), divided in its dispersive (*γ*^d^) and polar (*γ*^p^) components, and contact angle hysteresis (estimated from dynamic contact angles as the difference between the advancing and the receding angles) on both air and glass sides of the UV-cured resins and copolymers

Sample	Air side						Glass side					
	Static contact angle (°)		Surface energy (mN/m)			Contact angle hysteresis (°)	Static contact angle (°)		Surface energy (mN/m)			Contact angle hysteresis (°)
	${\theta}_{{\mathrm{H}}_20}$	${\theta}_{C_{16}{\mathrm{H}}_{34}}$	*γ*^d^	*γ*^p^	***γ***	CAH	${\theta}_{{\mathrm{H}}_20}$	${\theta}_{C_{16}{\mathrm{H}}_{34}}$	*γ*^d^	*γ*^p^	***γ***	CAH
TVE	56	12	27	20	47	25	53	7	27	22	49	39
PFPAE-EGVE + TVE	87	65	14	8	22	23	80	60	15	11	27	20
PFPAE-BGVE + TVE	95	69	13	5	18	23	90	66	14	7	21	17
PFPAE-DEGVE + TVE	83	63	14	10	24	20	73	62	15	16	31	21
TGE	58	5	28	18	46	43	51	5	23	28	51	34
PFPAE-MO + TGE	92	68	13	6	19	17	86	61	15	8	23	29
PFPAE-EO + TGE	101	70	12	3	15	11	80	63	14	11	25	24
PFPAE-PO + TGE	87	65	14	8	22	10	86	64	14	9	23	19

Testing the photopolymerized films made of the pure hydrogenated resins, both sides (i.e., both air and substrate sides) showed similar results: values displayed are common to polymers of medium polarity [23, 53]. Instead, the wettability of the fluorinated copolymers was significantly different, indicating a strong increase of water and oil repellency on both the air and the glass sides of the films. In particular, the hexadecane contact angle reached ca. 70° on the air side, and about 65° on the substrate side (more than 50° compared with the pure hydrogenated resins), as typically shown by highly fluorinated materials [14, 20]. The copolymer wettabilities are comparable with those exhibited by homologous fluorinated homopolymers, which show water contact angles ranging from 85° to 100°, and hexadecane contact angles in the range of 65–80° [24].

These results suggest that the external surface towards air is mostly composed by the fluorinated comonomer: this is in agreement with literature data on surface migration of fluoromonomers [14, 22, 54, 55]. However, with the content of fluoromonomer being quite high, there is no complete segregation of the PFPAE at the air surface; also, the surface in contact with glass is heterophasic and contains fluorine. Selective surface segregation at the air side happens when the fluoromonomer content is no more than 5 wt%: in these conditions the substrate side has a composition similar to the hydrogenated homopolymer [14, 22].

In agreement with the low wettabilities exhibited by these fluorinated copolymers, their surface energies (Table 4) are really low (around 20 mN/m): in particular, the decrease of the *γ* values is related to the depletion of the polar component (*γ*^p^) which shows a drop of about 15 mN/m for all the copolymers with respect to the hydrogenated resins (from the overall data collected in Table 4).

Surely, the surface proprieties do not only depend on the fluorine content (Table S1 of the Supporting Information). Correlations between the contact angle and surface energy values and the fine structure of the PFPAE comonomers can be discussed in terms of the molecular weight, the presence of monofunctional monomers, and the length and mobility of the fluorinated chain [56].

Also, dynamic contact angle and contact angle hysteresis (CAH) measurements (that is the difference between advancing and receding angles) were carried out on the resins and copolymers, on both sides of the UV-cured films (Fig. S7 of the Supporting Information): the data are collected in Table 4. The CAH is mainly related to the heterogeneity and/or roughness of the outermost layers, due to the presence of both hydrophobic and hydrophilic groups at the surface [53, 57].

As reported in Table 4, the CAH values of the copolymers containing PFPAEs are generally lower than those shown by the neat hydrogenated resins, suggesting that good anti-staining and self-cleaning properties can be obtained [14, 58].

As shown in Table 4, the hysteresis values of the PFPAE vinyl ether copolymers are higher than those exhibited by the PFPAE epoxide copolymers. Furthermore, a slight reduction of the hysteresis can be observed by increasing the length of the hydrogenated spacer between the fluorinated chain and the reactive end-group. However, the hysteresis behavior of the PFPAE copolymers is quite similar on both sides of the film (less than 5° and 10° of difference in the CAH values on the air and the glass sides for vinyl ethers and epoxides, respectively).

**Fig. 5:**
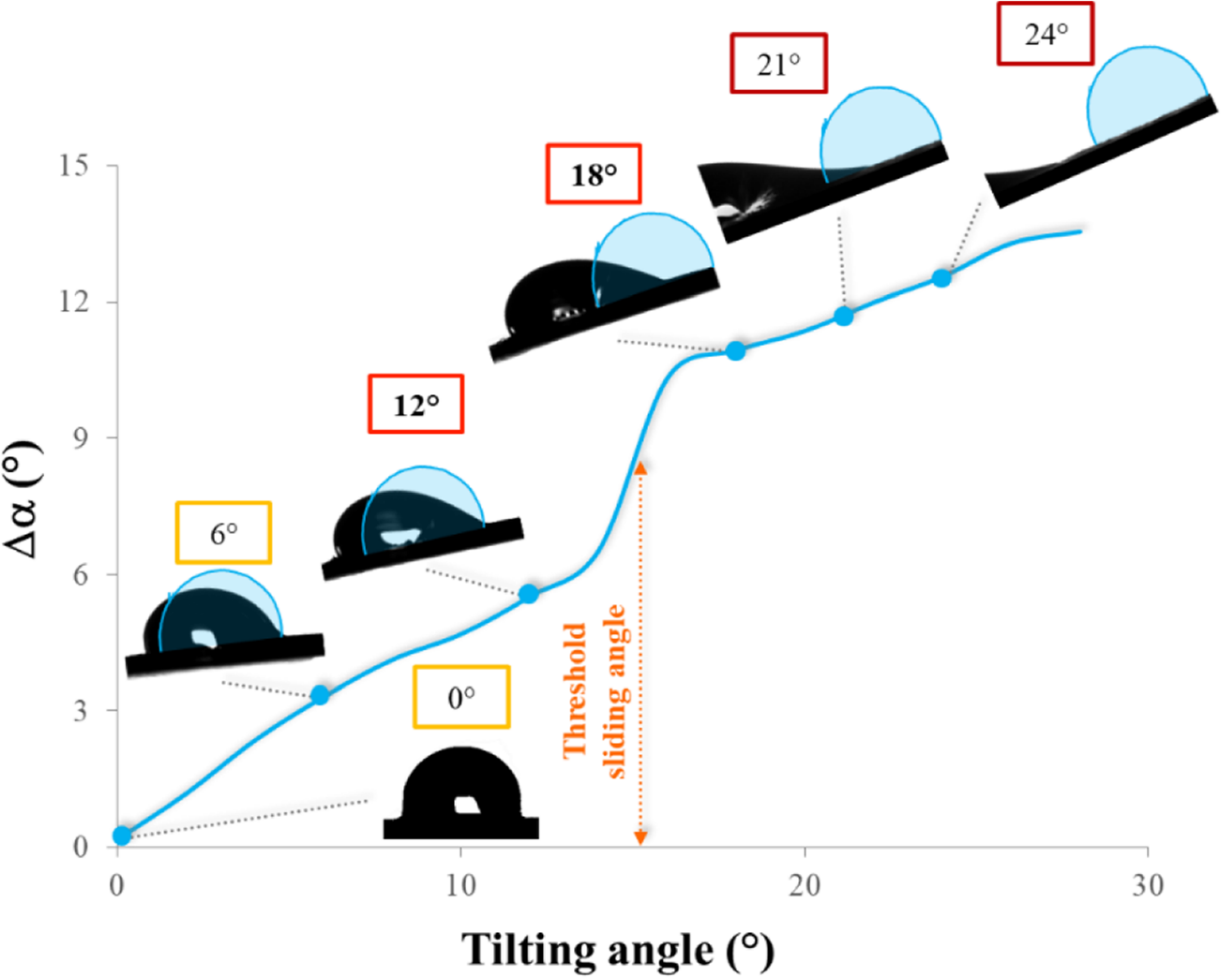
Δα (the difference between the advancing and the receding angles during tilting) as a function of the tilting angle, indicating the threshold sliding angle for water on the air side of a PFPAE-BGVE + TVE-cured film. The blue shadow represents the original shape of the water drop at a tilting angle of 0°

Also, sliding angle measurements were performed on the PFPAE copolymer surface. Tilting a surface on which a water drop is placed, an advancing and a receding contact angle can be measured. Increasing the tilting angle, the advancing contact angle increases, while the receding contact angle decreases. A plot of Δα (the difference between the advancing and the receding angles during tilting) versus the tilting angle can be drawn (Fig. 5) to better understand the value of the threshold sliding angle (i.e., the tilting angle at incipient droplet motion [59]).

Figure 5 shows that the threshold sliding of a 20 μL deionized water drop on a UV-cured film of PFPAE-BGVE + TVE is comprised between 15° and 17°, indicating that these copolymers are self-cleaning.

**Fig. 6:**
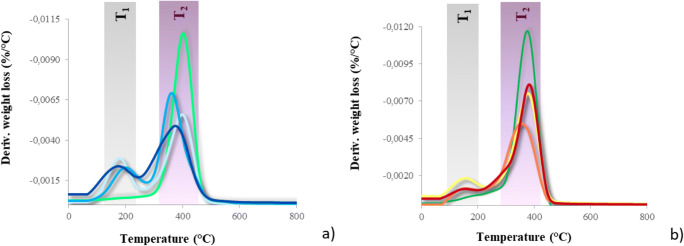
Derivative thermogravimetric (DTG) curves of the UV-cured networks. **a**  TVE,  PFPAE-EGVE + TVE,  PFPAE-BGVE + TVE, and  PFPAE-DEGVE + TVE; **b**  TGE,  PFPAE-MO + TGE,  PFPAE-EO + TGE, and  PFPAE-PO + TGE

TGA experiments were also performed, in inert atmosphere (N_2_), to evaluate the thermal stability of the photocured copolymers. The derivative thermogravimetric (DTG) curves are shown in Fig. 6. As it can be noticed, the thermal degradation of the PFPAE copolymers is characterized by two different main steps of weight loss, independently of the composition: these results can be attributed to a biphasic structure of the copolymers, as hypothesized above. The first degradation step begins at about 100 °C (*T*_1_, Fig. 6a, b), and the maximum degradation rate is around 200 °C. It was demonstrated that a facile and severe thermal degradation of PFPAE components can occur in the presence of Lewis acid (such as AlF_3_) [60] or even in presence of Al_2_O_3_ [61]: in these conditions, a complete degradation of fluorinated chains occurs above 180 °C [60, 61]. In our case, as the cationic polymerization requires the formation of superacids [1] that can still be present in the photocured networks, the thermal stability of the PFPAE-based copolymers has been adversely affected. The onset temperature of the PFPAE homopolymers degradation was found to be comparable with the one exhibited by fluorinated copolymers [24]. Concerning the second weight loss at ca. 300 °C (*T*_2_, Fig. 6a, b), it corresponds to the degradation of the hydrogenated moieties. The temperature corresponding to the loss of the 90% of the sample (*T*_90%_) is higher than 410 °C, and for temperatures higher than 600 °C, a complete degradation of the materials occurred and zero residue was detected.

However, observing both the temperature degradation data (collected in Table S2 of the Supporting Information) and the degrees of conversion of the photopolymerization reaction (Table 3), it seems that the copolymers showing a higher conversion are those more thermally resistant.

**Fig. 7:**
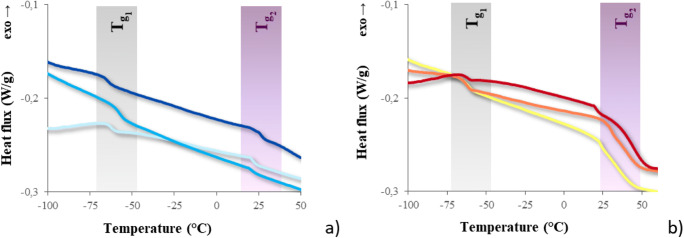
DSC thermograms of the photocured copolymers. **a**  PFPAE-EGVE + TVE,  PFPAE-BGVE + TVE, and  PFPAE-DEGVE + TVE; **b**  PFPAE-MO + TGE,  PFPAE-EO + TGE, and  PFPAE-PO + TGE

DSC thermograms are reported in Fig. 7. All the cured copolymers are fully amorphous and clearly exhibit two different glass transitions, due to the PFPAE phase and to the non-fluorinated phase, respectively. The low-temperature phase transition of the copolymers (*T*_g1_ ≅ − 65 °C; Fig. 7) is also present in the thermograms of the fluorinated homopolymers [24], and it is due to a fluorinated soft phase formed by the PFPAE segments. The second glass transition, which takes place at higher temperature (*T*_g2_ ≅ 20 °C for the vinyl ether copolymers and *T*_g2_ ≅ 35 °C for the epoxide copolymers), indicates the presence of a hard hydrogenated phase, made up of the alkyl resin and the hydrogenated segments of the fluorinated monomers. *T*_g2_ of the vinyl ether copolymers (Fig. 7a) has the same value of the glass transition of TVE (*T*_g_ ≅ 20 °C), as well as of the vinyl ether PFPAE homopolymers [24]. Whereas, *T*_g2_ of the epoxide copolymers (Fig. 7b) occurs at the same temperature of the phase transition of TGE (*T*_g_ ≅ 35 °C) but is slightly higher compared with the values showed by the epoxide-fluorinated homopolymers (occurring at ≅ 20 °C), probably due to the low conversion and the degree of phase separation [24].

The copolymers with PFPAE segments have been characterized also to evaluate their optical properties, in terms of refractive index (*n*). Measured refractive index values are collected in Table 5. Interestingly, two different values of refractive index of the bulk of each copolymer were observed, also varying the scanning wavelength. These results are another proof of the phase-separated structure of the copolymers. As expected, the PFPAE phase shows a lower refractive index (*n*_1_ ≅ 1.31) [11, 13] compared with the one exhibited by the hydrogenated moieties (*n*_2_ ≅ 1.51).

**Table 5:** Refractive index of the UV-cured resins and copolymers

Sample	Refractive index			
	*λ* = 633 nm		*λ* = 825 nm	
	*n*_1_	*n*_2_	*n*_1_	*n*_2_
TVE	–	1.508	–	1.504
PFPAE-EGVE + TVE	1.318	1.513	1.309	1.507
PFPAE-BGVE + TVE	1.323	1.516	1.301	1.510
PFPAE-DEGVE + TVE	1.324	1.515	1.322	1.508
TGE	–	1.508	–	1.503
PFPAE-MO + TGE	1.313	1.521	1.310	1.512
7PFPAE-EO + TGE	1.317	1.516	1.313	1.507
PFPAE-PO + TGE	1.312	1.515	1.313	1.508

The peculiarity of these photocured copolymers, in terms of unique optical properties, is given by the combination of two factors: the presence of two different refractive indexes in the same material and their phase-separated morphology. These characteristics can make the investigated PFPAE copolymers interesting materials for optical applications, especially for the fabrication of optical waveguide devices.

In conclusion, the results collected through the characterization of the films by multiple analyses (i.e., TGA, DSC, surface analysis, and optical measurements) suggest that the morphological structure of the UV-cured copolymers, as reported in [37], is quite complex. The external surface (towards air) is mostly composed of the lower refractive index component, the fluorinated comonomer (which is heterogeneous itself, as it contains PFPAE chains and alkyl spacers ended by oxirane or vinyl ether groups). The bulk is made of both fluorodomains and hydrogenated domains; however, it is richer in hydrogenated monomer (characterized by a higher refractive index), which constitutes the 75 wt% of the material. We expected a hard phase formed by the non-fluorinated resins and containing the hydrogenated spacers and the reactive end-groups of the PFPAE products. This hydrogenated hard phase of PFPAE copolymers can also contain fluorochains, in an amount depending on their molecular weight and distribution [37]. The opposite face of the film, built against glass, contains both hydrogenated and PFPAE units, with the latter of lower amount than in the bulk.

## Conclusions

New PFPAE derivatives, chain extended with different alkyl groups and functionalized with vinyl ether or epoxide end-groups, were employed as reactive comonomers to produce fluorinated copolymers exploiting the cationic photoinduced polymerization. The presence of fluorinated comonomers reduced the overall reactivity; however, the final conversions of the copolymeric systems were high (> 80%), as well as the gel content (> 80%).

The photocrosslinked copolymers showed marked hydrophobic and oleophobic surfaces, characterized by low hysteresis and low sliding angles. They were biphasic, as suggested by the presence of two different *T*_g_s observed in the DSC thermograms: one, at lower temperature, assigned to the softer fluorinated phase, while the other attributed to the hydrogenated domains. Two different values of refractive index were also observed, confirming a phase separated structure, in which, probably, the outermost layers are mostly made of the fluorinated comonomer (with lower refractive index), while the bulk is mainly composed of a hydrogenated network.

Thanks to these properties, the PFPAE copolymers can find applications as high-performance coatings, with anti-staining and self-cleaning properties, and eventually as polymer-based waveguides for optical applications.

## Electronic supplementary material

ESM 1 (PDF 857 kb).
