# Supplementary material for: Vinyl ethers and epoxides photoinduced copolymerization with perfluoropolyalkylether monomers
Source: Colloid Polym Sci. 2020 Sep 9;299(3):509–21. doi: 10.1007/s00396-020-04723-3 (PMC7952294; doi:10.1007/s00396-020-04723-3)
Supplement: Supplementary file 1 — (PDF 857 kb). [file 396_2020_4723_MOESM1_ESM.pdf]

Supporting Information

Vinyl ethers and epoxides photoinduced copolymerization with perfluoropolyalkylether monomers

Giuseppe Trusiano,[†,*] *Alessandra Vitale,*[†,*] *Céline Bonneaud,*[‡] *Diego Pugliese,*[†] *Sara Dalle Vacche*[†], *Christine Joly-Duhamel,*[‡] *Chadron M. Friesen,*[§] *Roberta Bongiovanni*[†]

[†] Department of Applied Science and Technology , Politecnico di Torino, Corso Duca degli Abruzzi 24, 10129 Torino, Italy

[‡] Institut Charles Gerhardt Montpellier , University of Montpellier, CNRS, ENSCM, Cedex 5, 34296 Montpellier, France

[§] Department of Chemistry, Trinity Western University, 7600 Glover Road, V2Y 1Y1 Langley, BC, Canada

* Corresponding authors: giuseppe.trusiano@polito.it ; alessandra.vitale@polito.it

List of Figures

Fig. S1 ATR FT-IR spectra of the PFPAE-EGVE + TVE copolymer: 3

Fig. S2 ATR FT-IR spectra of the PFPAE-BGVE + TVE copolymer: 3

Fig. S3 ATR FT-IR spectra of the PFPAE-DEGVE + TVE copolymer: 4

Fig. S4 ATR FT-IR spectra of the PFPAE-MO + TGE copolymer: 4

Fig. S5 ATR FT-IR spectra of the PFPAE-EO + TGE copolymer: 5

Fig. S6 ATR FT-IR spectra of the PFPAE-PO + TGE copolymer: 5

Fig. S7 Water contact angle hysteresis measurements, on air and glass sides, of the UV-cured copolymers: ... 7

List of Tables

Table S1 Number of repeat units, average molecular weight (M_n), difunctional content, (from ^{19}F-NMR spectra) of the functionalized PFPAE monomers, and composition details and fluorine content of the investigated copolymers. 6

Table S2 Degradation temperatures of the UV-cured hydrogenated resins and copolymers 7

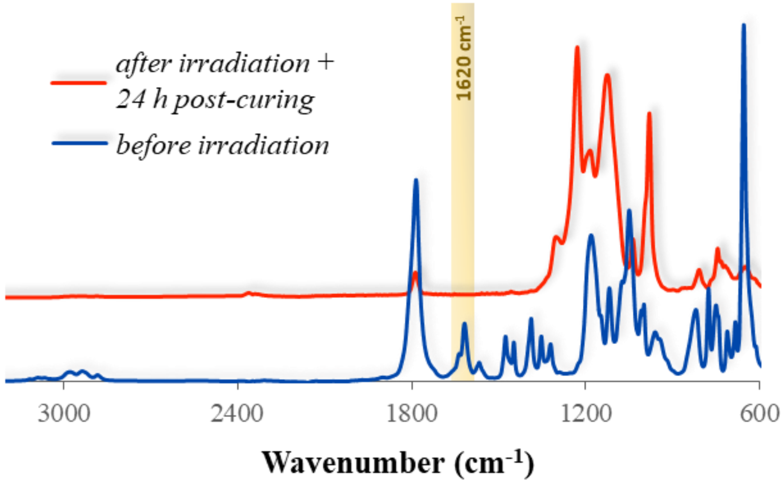

Fig. S1 ATR FT-IR spectra of the PFPAE-EGVE + TVE copolymer:

R*h*: peak ~**1780** cm^{-1} **C=O** bond; ~**1620** cm^{-1} **C=C**; peak ~**1100** cm^{-1} **C-O-C ethers**;

R*f*: peak ~**1240** cm^{-1} stretching **C-F** bond, and peak ~**1100** cm^{-1} **C-O-C ethers**;

Photoinitiator: peak ~**2950** cm^{-1} stretching C=**C-H**; peak ~**1780** cm^{-1} **C=O** bond; peak ~**1600-1320** cm^{-1} **C_6H_6** bonds; peak ~**1100** cm^{-1} **C-O-C ethers**

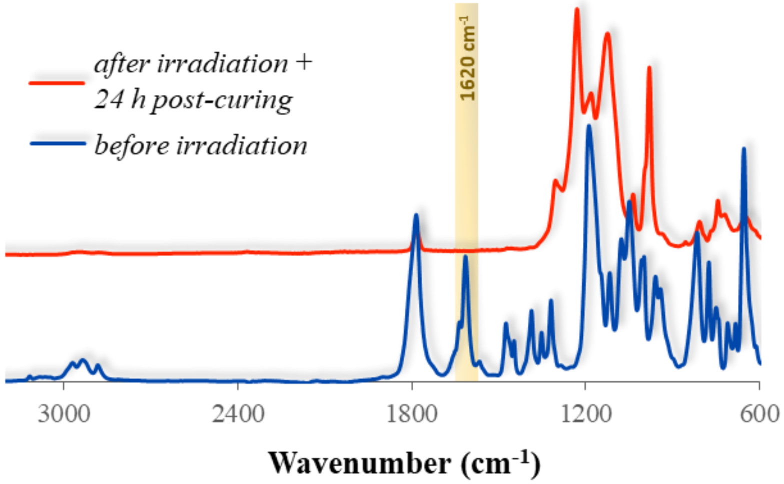

Fig. S2 ATR FT-IR spectra of the PFPAE-BGVE + TVE copolymer:

R*h*: peak ~**1780** cm^{-1} **C=O** bond; ~**1620** cm^{-1} **C=C**; peak ~**1100** cm^{-1} **C-O-C ethers**;

R*f*: peak ~**1240** cm^{-1} stretching **C-F** bond, and peak ~**1100** cm^{-1} **C-O-C ethers**;

Photoinitiator: peak ~**2950** cm^{-1} stretching C=**C-H**; peak ~**1780** cm^{-1} **C=O** bond; peak ~**1600-1320** cm^{-1} **C_6H_6** bonds; peak ~**1100** cm^{-1} **C-O-C ethers**

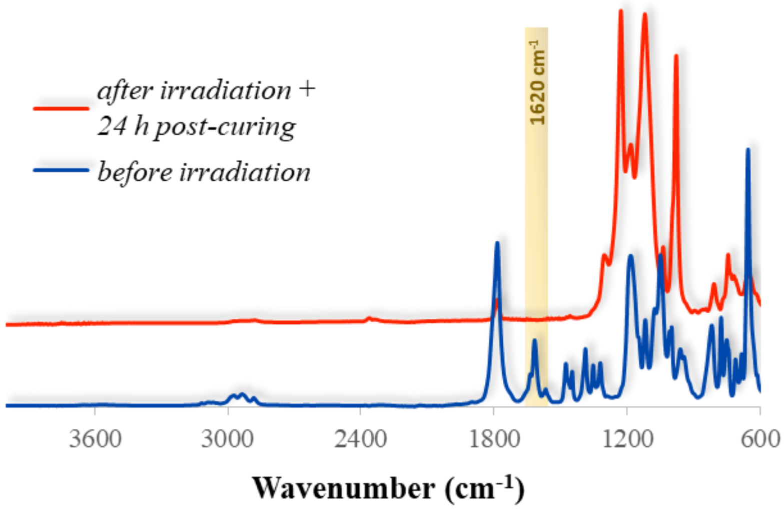

Fig. S3 ATR FT-IR spectra of the PFPAE-DEGVE + TVE copolymer:

R*h*: peak ~**1780** cm^{-1} **C=O** bond; ~**1620** cm^{-1} **C=C**; peak ~**1100** cm^{-1} **C-O-C ethers**;

R*f*: peak ~**1240** cm^{-1} stretching **C-F** bond, and peak ~**1100** cm^{-1} **C-O-C ethers**;

Photoinitiator: peak ~**2950** cm^{-1} stretching C=**C-H**; peak ~**1780** cm^{-1} **C=O** bond; peak ~**1600-1320** cm^{-1} **C_6H_6** bonds; peak ~**1100** cm^{-1} **C-O-C ethers**

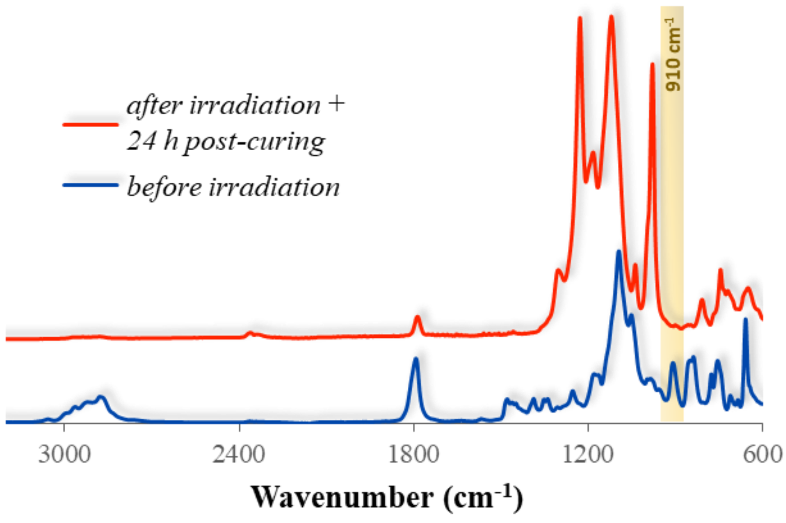

Fig. S4 ATR FT-IR spectra of the PFPAE-MO + TGE copolymer:

R*h*: peak ~**1780** cm^{-1} **C=O** bond; ~**1620** cm^{-1} **C=C**; peak ~**1100** cm^{-1} **C-O-C ethers**; peak ~**910** cm^{-1} **epoxides**;

R*f*: peak ~**1240** cm^{-1} stretching **C-F** bond, and peak ~**1100** cm^{-1} **C-O-C ethers**;

Photoinitiator: peak ~**2950** cm^{-1} stretching C=**C-H**; peak ~**1780** cm^{-1} **C=O** bond; peak ~**1600-1320** cm^{-1} **C_6H_6** bonds; peak ~**1100** cm^{-1} **C-O-C ethers**

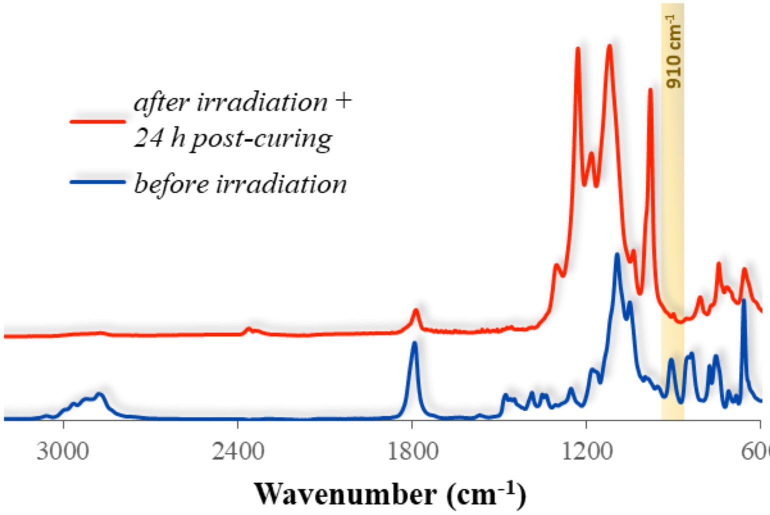

Fig. S5 ATR FT-IR spectra of the PFPAE-EO + TGE copolymer:

R$_h$: peak ~**1780** cm^{-1} **C=O** bond; ~**1620** cm^{-1} **C=C**; peak ~**1100** cm^{-1} **C-O-C ethers**; peak ~**910** cm^{-1} **epoxides**;

R$_f$: peak ~**1240** cm^{-1} stretching **C-F** bond, and peak ~**1100** cm^{-1} **C-O-C ethers**;

Photoinitiator: peak ~**2950** cm^{-1} stretching C=**C-H**; peak ~**1780** cm^{-1} **C=O** bond; peak ~**1600-1320** cm^{-1} **C_6H_6** bonds; peak ~**1100** cm^{-1} **C-O-C ethers**

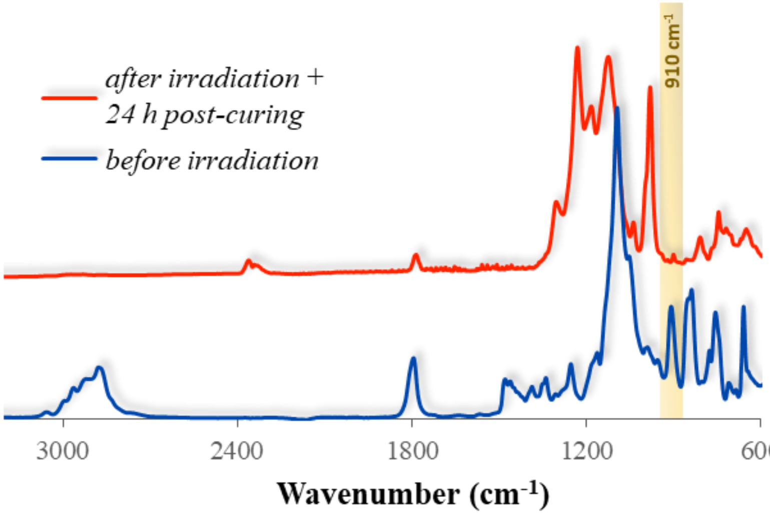

Fig. S6 ATR FT-IR spectra of the PFPAE-PO + TGE copolymer:

R$_h$: peak ~**1780** cm^{-1} **C=O** bond; ~**1620** cm^{-1} **C=C**; peak ~**1100** cm^{-1} **C-O-C ethers**; peak ~**910** cm^{-1} **epoxides**;

R$_f$: peak ~**1240** cm^{-1} stretching **C-F** bond, and peak ~**1100** cm^{-1} **C-O-C ethers**;

Photoinitiator: peak ~**2950** cm^{-1} stretching C=**C-H**; peak ~**1780** cm^{-1} **C=O** bond; peak ~**1600-1320** cm^{-1} **C_6H_6** bonds; peak ~**1100** cm^{-1} **C-O-C ethers**

Table S1 Number of repeat units, average molecular weight (M_n), difunctional content, (from ^{19}F-NMR spectra) of the functionalized PFPAE monomers, and composition details and fluorine content of the investigated copolymers.

Copolymer	*m*	PFPAE molecular weight (g/mol)	PFPAE difunctional content (mol%)	PFPAE/Resin weight ratio	F content in copolymer (wt%)	F content in copolymer (mol%)
PFPAE-EGVE + TVE	6	1740	56	0.32	18.45	0.97
PFPAE-BGVE + TVE	8	2130	88	0.32	18.93	1.00
PFPAE-DEGVE + TVE	7	2000	43	0.32	16.46	0.87
PFPAE-MO + TGE	12	2720	63	0.32	17.06	0.90
PFPAE-EO + TGE	8	2130	41	0.32	16.06	0.85
PFPAE-PO + TGE	10	2530	58	0.32	16.08	0.85

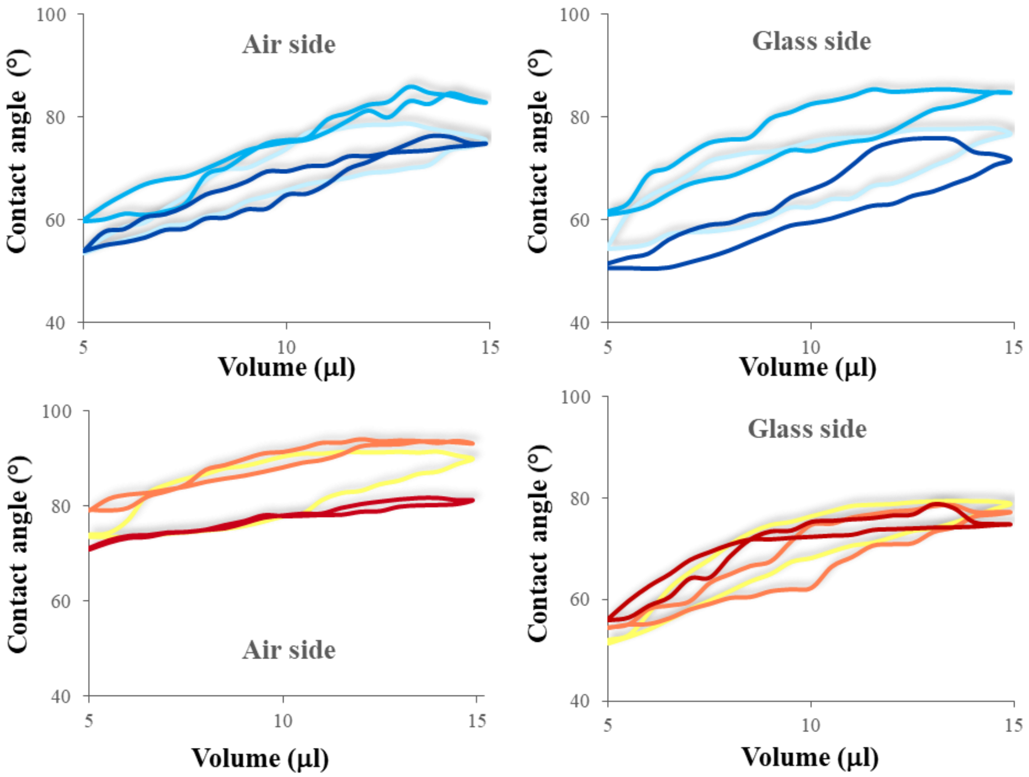

Fig. S7 Water contact angle hysteresis measurements, on air and glass sides, of the UV-cured copolymers:

PFPAE-EGVE + TVE, PFPAE-BGVE + TVE, PFPAE-DEGVE + TVE,

PFPAE-MO + TGE, PFPAE-EO + TGE, PFPAE-PO + TGE.

Table S2 Degradation temperatures of the UV-cured hydrogenated resins and copolymers

System	T_{onset} (°C)	T_{max1} (°C)	T_{max2} (°C)	$T_{90\%}$ (°C)
TVE	188	-	398	428
PFPAE-EGVE + TVE	137	181	397	427
PFPAE-BGVE + TVE	150	192	366	429
PFPAE-DEGVE + TVE	136	182	375	429
TGE	185	-	378	405
PFPAE-MO + TGE	130	156	381	413
PFPAE-EO + TGE	132	159	361	410
PFPAE-PO + TGE	140	159	385	413
